# Endovascular Therapy Versus Open Surgery for Common Femoral Artery Atherosclerotic Occlusive Disease: A Systematic Review and Meta-Analysis

**DOI:** 10.3390/jcm15145353

**Published:** 2026-07-08

**Authors:** Chuwen Chen, Yiyuan Li, Jing Hu, Lijia Wei, Hankui Hu, Bin Huang, Xiyang Chen

**Affiliations:** 1 Division of Vascular Surgery, Department of General Surgery, West China Hospital, Sichuan University, Chengdu 610041, China; chenchuwen@scu.edu.cn (C.C.); liyiyuan@stu.scu.edu.cn (Y.L.); weilijia@stu.scu.edu.cn (L.W.); huhankui@wchscu.cn (H.H.); huangbin@wchscu.cn (B.H.); 2 Department of Health Management Centre, West China Fourth Hospital, Sichuan University, Chengdu 610041, China; hxfsyhujing@163.com

**Keywords:** common femoral artery, peripheral arterial disease, atherosclerotic occlusive, endovascular therapy, open surgery, meta-analysis

## Abstract

The optimal revascularization strategy for common femoral artery (CFA) atherosclerotic occlusive disease remains debated. This systematic review and meta-analysis compared perioperative and long-term outcomes of endovascular therapy (EVT) and open surgery (OS). **Methods**: PubMed, Embase, Web of Science, the Cochrane Library, and ClinicalTrials.gov were searched through 15 March 2026 for comparative studies of EVT versus OS in CFA atherosclerotic occlusive disease. Outcomes included perioperative morbidity, wound complications, hospital stay, patency, reintervention, major amputation, and survival. Time-to-event data were pooled as hazard ratios (HRs), reported or reconstructed from Kaplan-Meier curves. Risk of bias and evidence certainty were assessed. **Results**: Eleven studies with 2835 patients were included. Compared with OS, EVT reduced 30-day morbidity (OR, 0.34; 95% CI: 0.26–0.44), wound complications (OR, 0.14; 95% CI: 0.09–0.23), surgical-site infections, lymphatic complications, myocardial infarction, and hospital stay (mean difference, −4.68 days; 95% CI: −5.49 to −3.86). Distal embolization increased after EVT (OR, 2.45; 95% CI: 1.28–4.70). Follow-up major amputation was higher after EVT in event-rate analyses (OR, 2.42; 95% CI: 1.19–4.93), although amputation-free survival was similar (HR, 1.01; 95% CI: 0.83–1.23). EVT had higher hazards of loss of primary patency (HR, 1.72), loss of secondary patency (HR, 2.03), and reintervention (HR, 1.51). **Conclusions**: EVT offers fewer early complications and shorter hospitalization, whereas OS provides more durable patency and fewer reinterventions. Given observational, heterogeneous evidence, treatment should be individualized.

## 1. Introduction

The common femoral artery (CFA) is a critical anatomic junction in the lower-extremity arterial system, connecting the iliac artery with the superficial femoral artery and the profunda femoris artery. Accordingly, atherosclerotic occlusive disease involving the CFA can directly compromise lower-limb perfusion. With population aging and the increasing prevalence of metabolic disorders, CFA atherosclerotic occlusive disease has become an important challenge in the management of peripheral arterial disease (PAD) [1]. For decades, open surgery (OS) has been regarded as the standard treatment for CFA atherosclerotic occlusive disease because it can achieve durable vessel patency through direct exposure of the diseased artery followed by endarterectomy, patch angioplasty, or bypass reconstruction [1,2]. However, OS is invasive and may be associated with postoperative wound and lymphatic fistula, particularly in elderly patients and those with multiple comorbidities. In recent years, endovascular therapy (EVT) has advanced rapidly, and minimally invasive techniques such as balloon angioplasty, stent implantation, atherectomy, and drug-coated balloon (DCB) angioplasty have been increasingly used for PAD [3]. EVT offers potential advantages, including reduced surgical trauma, accelerated recovery, shorter hospitalization, and a lower incidence of perioperative wound-related complications. Accordingly, EVT may be a more appropriate treatment option, especially for frail, sarcopenic, or elderly patients, as well as those with multiple comorbidities [4,5]. Nevertheless, the CFA is located beneath the inguinal ligament and is exposed to repetitive flexion-related mechanical stress. Implanted stents may therefore be vulnerable to deformation, fracture, neointimal hyperplasia, and restenosis. In addition, because the CFA is a bifurcation site, endovascular manipulation may compromise the orifice of the profunda femoris artery and increase the risk of distal embolization. Current guidelines generally continue to favor open surgical treatment for appropriately selected patients with CFA occlusive disease and discourage routine stent implantation in this anatomic segment, although the level of supporting evidence remains limited [1].

Although previous systematic reviews [6,7,8] have compared EVT with OS for CFA disease, several contemporary studies [9,10,11,12,13] have since been published, including reports evaluating newer endovascular strategies such as interwoven nitinol stents [9], drug-coated balloons [14], and atherectomy-assisted interventions [12]. In addition, prior syntheses have largely relied on fixed-time patency estimates rather than time-to-event analyses. This systematic review and meta-analysis therefore aimed to provide an updated comparison of EVT versus OS for CFA atherosclerotic occlusive disease, incorporating contemporary literature, hazard ratio-based analyses of patency and reintervention, and updated estimates of perioperative morbidity.

## 2. Methods

This systematic review and meta-analysis synthesized published aggregate data and did not involve direct contact with participants or collection of identifiable individual-level data; therefore, institutional review board approval and informed consent were not required. The protocol was registered in PROSPERO (CRD420251126659), and the study was conducted in accordance with the Preferred Reporting Items for Systematic Reviews and Meta-Analyses (PRISMA) statement (Supplementary Table S5) [15].

### 2.1. Search Strategy and Literature Screening

Two independent investigators (C.W.C. and J.H.) independently performed a systematic literature search of PubMed, Embase, Web of Science, the Cochrane Library, and ClinicalTrials.gov from database inception to 15 March 2026. The search strategy combined controlled vocabulary terms (e.g., MeSH in PubMed and Emtree in Embase) and free-text keywords related to “atherosclerosis,” “peripheral artery disease,” “arterial occlusive disease,” “intermittent claudication,” “limb ischemia,” “stenosis,” “occlusion,” “endovascular,” “angioplasty,” “balloon,” “stent,” “surgery,” “endarterectomy,” and “common femoral artery”. No initial restriction on study design was applied during the electronic search in order to maximize sensitivity. The reference lists of all included studies and relevant reviews were manually screened to identify additional eligible records. When multiple publications reported overlapping patient populations, the most complete or most recent dataset was included.

### 2.2. Inclusion and Exclusion Criteria

#### Inclusion Criteria

Studies were included if they met the following criteria: (1) studies directly compared EVT with OS for CFA atherosclerotic occlusive disease; (2) atherosclerotic stenosis or occlusive disease involving the common femoral artery (CFA), including lesions extending to the femoral bifurcation, profunda femoris artery origin, or proximal superficial femoral artery when CFA-specific data were available; (3) EVT included balloon angioplasty, stent implantation, drug-coated balloon angioplasty, atherectomy, including directional, rotational, or hybrid atherectomy, or other endovascular techniques; (4) OS included common femoral endarterectomy, patch angioplasty, bypass surgery, or other open reconstructive procedures; (5) at least one relevant outcome was reported, including primary patency, secondary patency, target lesion revascularization, perioperative complications, limb salvage, major amputation, mortality, or another clinically relevant endpoint; (6) the article was available as a full-text publication in English.

### 2.3. Exclusion Criteria

Studies were excluded if they met any of the following criteria: (1) the underlying pathology was not atherosclerotic, including traumatic arterial injury, iatrogenic pseudoaneurysm, arteriovenous fistula, vasculitis, radiation-induced arterial disease, infection, congenital vascular malformation, acute embolic occlusion, or acute thrombotic occlusion without evidence of chronic atherosclerotic disease; (2) studies were excluded if data on common femoral artery disease could not be extracted separately from data on other arterial segments; (3) case reports, case series, reviews, editorials, conference abstracts, technical notes, letters, or studies without original data; (4) studies with unclear intervention definitions or without a direct comparison between EVT and OS; (5) insufficient data to estimate effect sizes or extract relevant outcome measures; (6) duplicate publications or overlapping datasets, in which case only the most comprehensive study was included.

### 2.4. Outcomes Definitions

For hybrid procedures, group assignment was determined by the treatment applied to the CFA component. Studies in which the CFA lesion was treated with endarterectomy, patch angioplasty, bypass, or another open reconstructive procedure were classified as OS, whereas those in which the CFA lesion was treated with balloon angioplasty, stenting, drug-coated balloon angioplasty, atherectomy, or another endovascular technique were classified as EVT. Perioperative morbidity/mortality was defined as any complication/death occurring within 30 days after the index procedure. Primary patency was defined as uninterrupted patency of the treated segment without restenosis, occlusion, or any additional endovascular or surgical reintervention. Secondary patency was defined as patency of the treated vessel maintained after one or more reinterventions performed to restore blood flow following initial loss of primary patency. Major amputation was defined as any amputation performed above the ankle of the affected limb during follow-up.

### 2.5. Data Extraction and Quality Assessment

#### Data Extraction

Two reviewers (B.H. and H.R.H.) independently extracted and synthesized data from the included studies, including baseline patient characteristics, procedural outcomes, and clinical outcomes. Any uncertainties or disagreements were resolved through consultation with a third reviewer (X.Y.C.) until consensus was reached. Clinically relevant variables included the crude number of events, when available, event rates in each cohort, the type and magnitude of the effect estimate, corresponding confidence intervals, severity of lower limb ischemia, and follow-up duration. Hazard ratios (HRs) and corresponding standard errors were extracted directly when reported. When HRs were not provided, they were reconstructed from Kaplan-Meier curves using Engauge Digitizer and the methods described by Tierney et al. [16]. All HRs were harmonized so that values greater than 1 favored OS for patency-related outcomes by indicating a higher hazard of loss of patency or reintervention in the EVT group.

### 2.6. Quality Assessment

Risk of bias was independently assessed by two reviewers using design-specific tools. The Risk Of Bias In Non-randomised Studies of Interventions (ROBINS-I) [17] was applied to observational studies, including retrospective and prospective cohort designs, while randomized controlled trials (RCTs) were evaluated using the Cochrane Risk of Bias 2 (RoB 2) [18] tool. Disagreements were resolved by consensus, with third-party adjudication when necessary.

### 2.7. Certainty of Evidence

The certainty of evidence for key outcomes was assessed using the Grading of Recommendations Assessment, Development and Evaluation (GRADE) framework. Outcomes considered clinically important included 30-day perioperative morbidity, wound complications, surgical-site infection, lymphatic complications, distal embolization, perioperative myocardial infarction, hospital length of stay, follow-up major amputation, loss of primary patency, loss of secondary patency, and reintervention. Certainty was rated as high, moderate, low, or very low according to risk of bias, inconsistency, indirectness, imprecision, and publication bias. Because the evidence base included both randomized controlled trials and observational studies, certainty ratings were judged according to the predominant source of evidence for each outcome and were downgraded when pooled estimates were primarily derived from nonrandomized studies, studies at moderate risk of bias, outcomes with substantial heterogeneity, low event rates, or reconstructed time-to-event data.

### 2.8. Statistical Analysis

Statistical analyses were performed using Review Manager (RevMan), version 5.4 (The Cochrane Collaboration, 2020). Continuous variables were expressed as means and standard deviations, whereas categorical variables were reported as percentages. For studies reporting continuous outcomes as medians and ranges, the corresponding means and standard deviations were estimated using the method described by Wan et al. [19]. Publication bias was assessed by funnel plot analysis when at least 10 studies were available. Statistical heterogeneity was evaluated using Cochran’s Q test and quantified with the I^2^ statistic, with I^2^ > 50% indicating substantial heterogeneity. A random-effects model was applied when significant heterogeneity was present. A *p* value < 0.05 was considered statistically significant.

## 3. Results

### 3.1. Characteristics of the Included Studies and Patient Demographics

**Figure 1:**
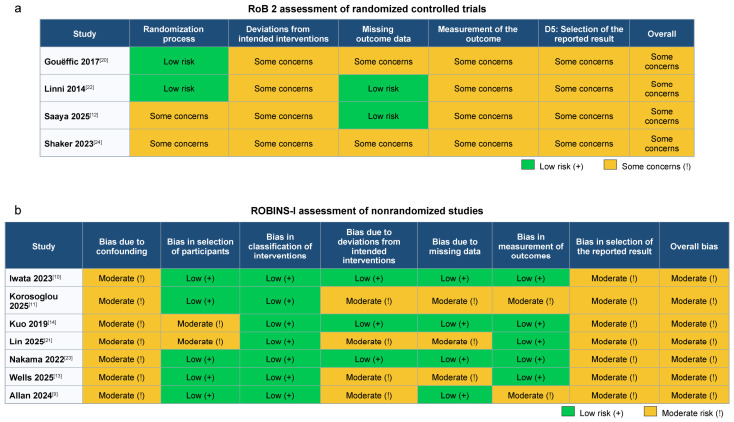
Risk-of-bias assessment of included studies. (**a**): RoB 2 assessment of randomized controlled trials; (**b**): ROBINS-I assessment of observational studies [9,10,11,12,13,14,20,21,22,23,24].

**Figure 2:**
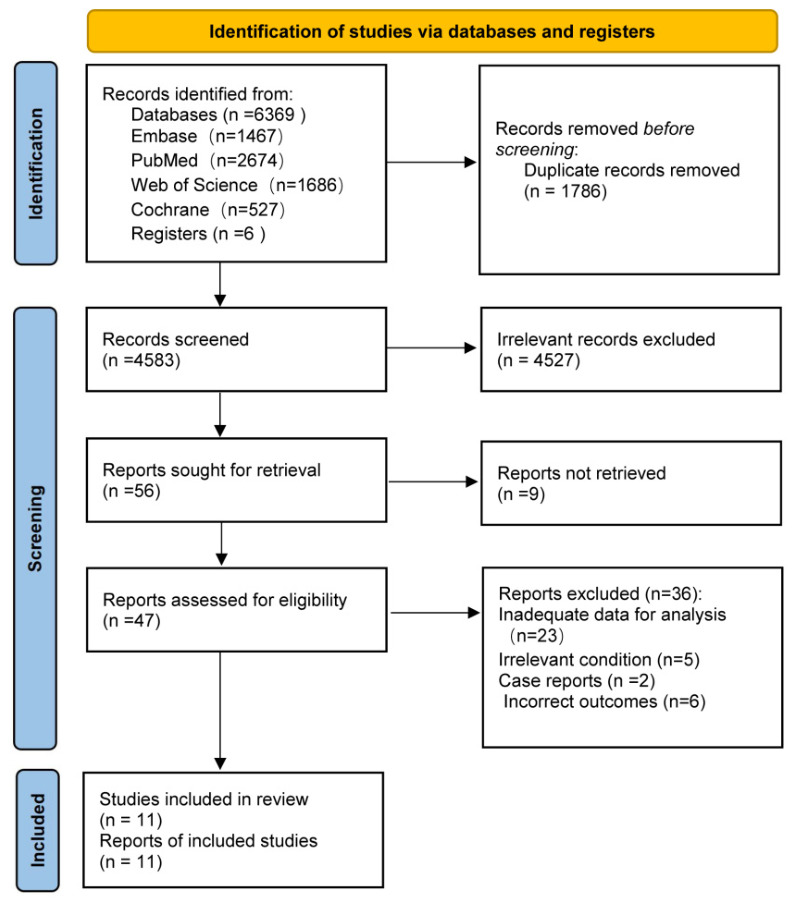
PRISMA flow diagram of study selection. PRISMA: preferred reporting items for systematic reviews and meta-analyses.

A total of 11 studies were included [9,10,11,12,13,14,20,21,22,23,24], comprising four RCTs [12,20,22,24] and seven observational studies (including two propensity score-matched analyses) [9,10,11,13,14,21,23]. Four included studies [11,12,21,24] reported hybrid procedures; however, because the CFA component was treated with common femoral endarterectomy in all cases, these studies were classified into the OS group. All seven retrospective studies demonstrated moderate risk of bias based on ROBINS-I assessment (Table 1). All four RCTs [12,20,22,24] assessed with RoB 2 were judged as having “some concerns” for overall risk of bias (Figure 1). Figure 2 illustrates the literature screening process for the 11 included studies, while Table 1 presents the baseline characteristics of these studies.

The 11 studies [9,10,11,12,13,14,20,21,22,23,24] encompassed a total of 2835 patients with CFA atherosclerotic occlusive disease. Compared with the OS group, the EVT group had a slightly higher preoperative ankle-brachial index (ABI) value (MD = 0.02; 95% CI: 0.01–0.04; *p* = 0.002; I^2^ = 27%; Table 2), although no significant differences were observed in the RCT, cohort, or hybrid subgroup analyses (Supplementary Tables S1–S3). The prevalence of coronary artery disease was also higher in the EVT group (OR, 1.26; 95% CI: 1.07–1.48; *p* = 0.005; I^2^ = 9%; Table 2), with similar findings observed only in the cohort subgroup (Supplementary Table S1). In addition, hyperlipidemia was more common in the EVT group than in the OS group (OR = 1.43; 95% CI: 1.14–1.79; *p* = 0.002; I^2^ = 0%; Table 2), with consistent findings in the cohort and hybrid subgroup analyses where applicable (Supplementary Tables S1 and S3). No significant differences were observed between the two groups in age (MD = 0.50; 95% CI: −0.41–1.42; *p* = 0.28; I^2^ = 43%; Table 2), diabetes (OR = 1.11; 95% CI: 0.85–1.45; *p* = 0.44; I^2^ = 48%; Table 2), gender (OR = 0.86; 95% CI: 0.65–1.14; *p* = 0.30; I^2^ = 45%; Table 2), hypertension (OR = 1.07; 95% CI: 0.81–1.40; *p* = 0.64; I^2^ = 0%; Table 2), chronic renal insufficiency (OR = 1.21; 95% CI: 0.98–1.49; *p* = 0.08; I^2^ = 0%; Table 2), rest pain (OR = 1.18; 95% CI: 0.86–1.61; *p* = 0.31; I^2^ = 26%; Table 2), chronic limb-threatening ischemia (OR = 0.88; 95% CI: 0.56–1.39; *p* = 0.58; I^2^ = 63%; Table 2), hemodialysis (OR = 0.97; 95% CI: 0.67–1.41; *p* = 0.87; I^2^ = 0%; Table 2), Rutherford stage > 3 (OR = 0.89; 95% CI: 0.61–1.31; *p* = 0.55; I^2^ = 63%; Table 2), history of stroke (OR = 0.97; 95% CI: 0.75–1.25; *p* = 0.81; I^2^ = 0%; Table 2), proportion of current smokers (OR = 0.80; 95% CI: 0.61–1.05; *p* = 0.11; I^2^ = 40%; Table 2), calcification ≥ 50% (OR = 0.89; 95% CI: 0.70–1.13; *p* = 0.34; I^2^ = 58%; Table 2), or claudication symptoms (OR = 0.93; 95% CI: 0.69–1.25; *p* = 0.63; I^2^ = 25%; Table 2).

### 3.2. Postoperative 30-Day Outcomes

**Figure 3:**
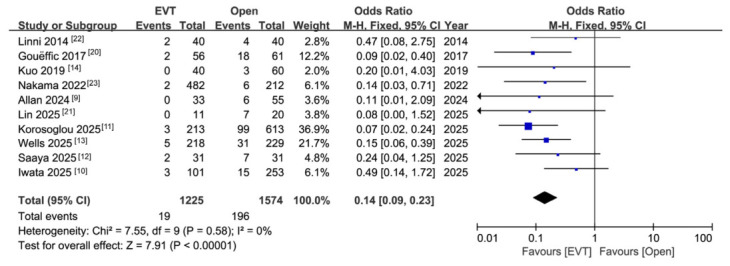
Forest plot of the meta-analysis of perioperative morbidity between EVT and open surgery. M-H: Mantel–Haenszel; CI: confidence interval [9,10,11,12,13,14,20,21,22,23].

**Figure 4:**
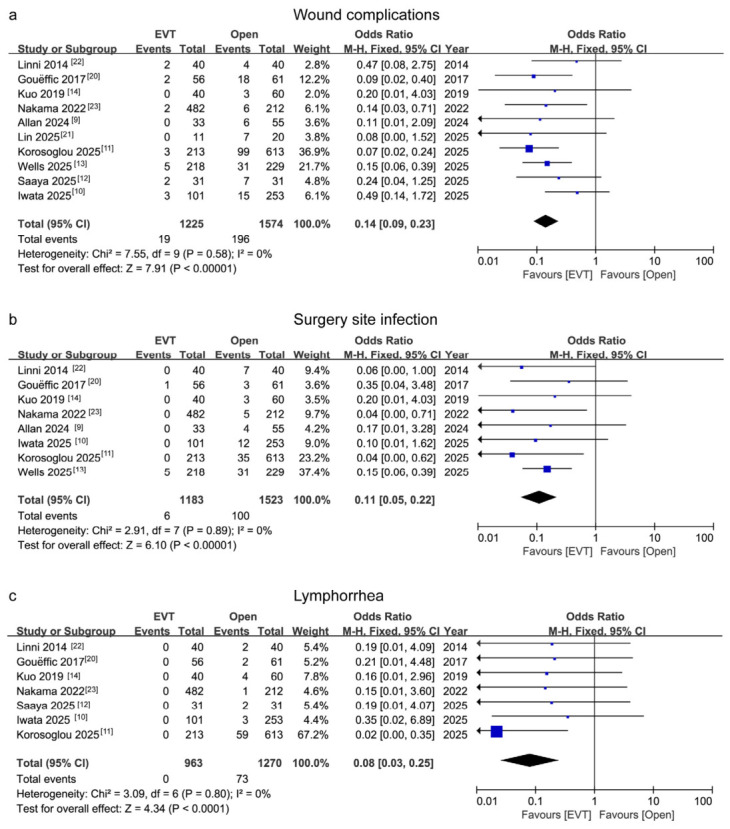
(**a**): Forest plot of the meta-analysis of wound complications between EVT and open surgery. (**b**): Forest plot of the meta-analysis of surgical-site infection between EVT and open surgery. (**c**): Forest plot of the meta-analysis of lymphatic fistula between EVT and open surgery. M-H: Mantel–Haenszel; CI: confidence interval [9,10,11,12,13,14,20,21,22,23].

**Figure 5:**
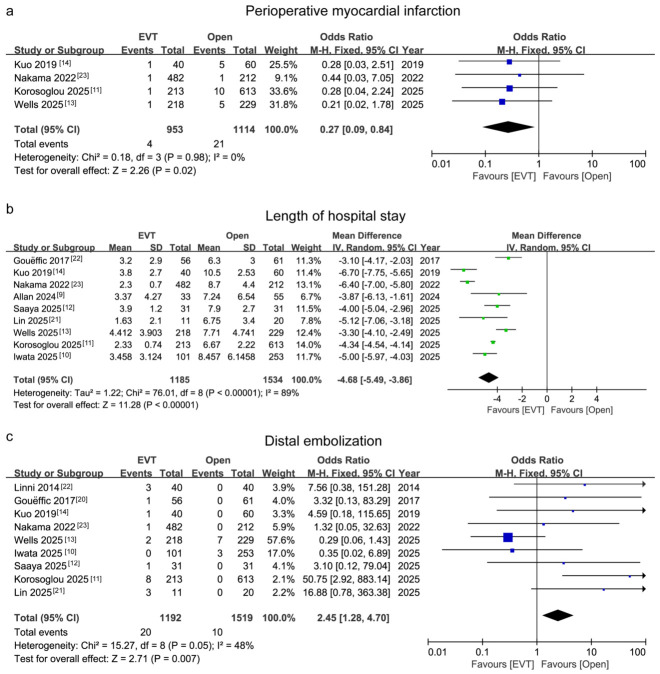
(**a**): Forest plot of the meta-analysis of perioperative myocardial infarction between EVT and open surgery. (**b**): Forest plot of the meta-analysis of length of hospital stay between EVT and open surgery. (**c**): Forest plot of the meta-analysis of distal embolization between EVT and open surgery. M-H, Mantel–Haenszel; CI, confidence interval [9,10,11,12,13,14,20,21,22,23].

Perioperative morbidity was reported in 10 studies [9,10,11,12,14,20,21,22,23,24] and was significantly lower after EVT than after OS (EVT vs. OS: 8.00% vs. 22.81%; OR = 0.34; 95% CI: 0.26–0.44; *p* < 0.001; I^2^ = 0%; Figure 3). Similarly, EVT was associated with significantly lower rates of wound complications across 10 studies [9,10,11,12,14,20,21,22,23,24] (EVT vs. OS: 1.55% vs. 12.45%; OR = 0.14; 95% CI: 0.09–0.23; *p* < 0.001; I^2^ = 0%; Figure 4a), surgical-site infection across eight studies [9,10,11,13,14,20,22,23] (EVT vs. OS: 0.51% vs. 6.57%; OR = 0.11; 95% CI: 0.05–0.22; *p* < 0.001; I^2^ = 0%; Figure 4b) and lymphatic fistula formation across seven studies [10,11,13,14,20,22,23] (EVT vs. OS: 0.00% vs. 5.75%; OR = 0.08; 95% CI: 0.03–0.25; *p* < 0.001; I^2^ = 0%; Figure 4c). Findings were consistent in the cohort and hybrid subgroup analyses where applicable (Supplementary Tables S1 and S3). Perioperative myocardial infarction was reported in three studies [11,13,14] and was less frequent after EVT compared with the OS group (EVT vs. OS: 0.21% vs. 2.22%; OR = 0.17; 95% CI: 0.04–0.72; *p* = 0.02; I^2^ = 0%; Figure 5a). Nine studies [9,10,11,12,13,14,20,21,23] presented hospital length of stay data and synthesized analysis indicated that the EVT group had significantly shorter hospital stays than the OS group (EVT vs. OS: mean difference = −4.68 days; 95% CI: −5.49 to −3.86; *p* < 0.001; I^2^ = 89%; Figure 5b). Consistent findings were observed in the RCT, cohort study, and hybrid subgroup analyses (Supplementary Tables S1–S3). Nine studies [10,11,12,13,14,20,21,22,23] documented distal embolization events and meta-analysis revealed that the EVT group experienced significantly higher rates of distal embolization compared with the OS group (EVT vs. OS: 1.68% vs. 0.66%; OR = 2.45; 95% CI: 1.28–4.70; *p* = 0.007; I^2^ = 48%; Figure 5c). No significant differences were observed between the two groups in perioperative mortality (OR = 1.16; 95% CI: 0.54–2.46; *p* = 0.70; I^2^ = 10%; Table 3), reoperation (OR = 0.51; 95% CI: 0.17–1.52; *p* = 0.23; I^2^ = 56%; Table 3), new hemodialysis requirement (OR = 1.21; 95% CI: 0.66–2.22; *p* = 0.53; I^2^ = 0%; Table 3), major amputation (OR = 0.78; 95% CI: 0.31–1.99; *p* = 0.61; I^2^ = 36%; Table 3), hematoma formation (OR = 0.71; 95% CI: 0.39–1.31; *p* = 0.28; I^2^ = 51%; Table 3), stroke (OR = 0.97; 95% CI: 0.36–2.59; *p* = 0.95; I^2^ = 0%; Table 3), or postoperative ankle brachial index (MD = −0.04; 95% CI: −0.11 to 0.03; *p* = 0.29; I^2^ = 96%; Table 3).

### 3.3. Follow-Up Outcomes

**Figure 6:**
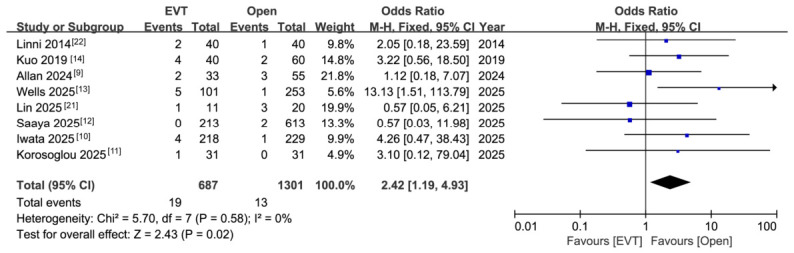
Forest plot of the meta-analysis of follow-up major amputation between EVT and open surgery. M-H, Mantel–Haenszel; CI, confidence interval [9,10,11,12,13,14,21,22].

Eight studies [9,10,11,12,13,14,21,22] reported major amputation outcomes during follow-up and pooled analysis demonstrated that the EVT group had a significantly higher rate of major amputation events compared with the OS group (EVT vs. OS: 2.77% vs. 1.00%; OR = 2.42; 95% CI: 1.19–4.93; *p* = 0.02; I^2^ = 0%; Figure 6). Consistent findings were observed in the cohort study subgroup analyses (Supplementary Table S1). However, the time-to-event analysis for amputation-free survival did not show a significant difference between groups. Seven studies [10,11,13,14,21,22,23] reported mortality during follow-up, and no significant difference was identified between the two groups (OR = 1.48; 95% CI: 0.75–2.95; *p* = 0.26; I^2^ = 38%; Table 4). Similarly, pooled analyses showed no significant between-group differences in myocardial infarction (OR = 0.56; 95% CI: 0.12–2.63; *p* = 0.46; I^2^ = 53%; Table 4), stroke (OR = 1.06; 95% CI: 0.46–2.46; *p* = 0.89; I^2^ = 34%; Table 4), or reintervention (OR = 1.22; 95% CI: 0.87–1.71; *p* = 0.26; I^2^ = 0%; Table 4).

### 3.4. Time-to-Event Outcomes

**Figure 7:**
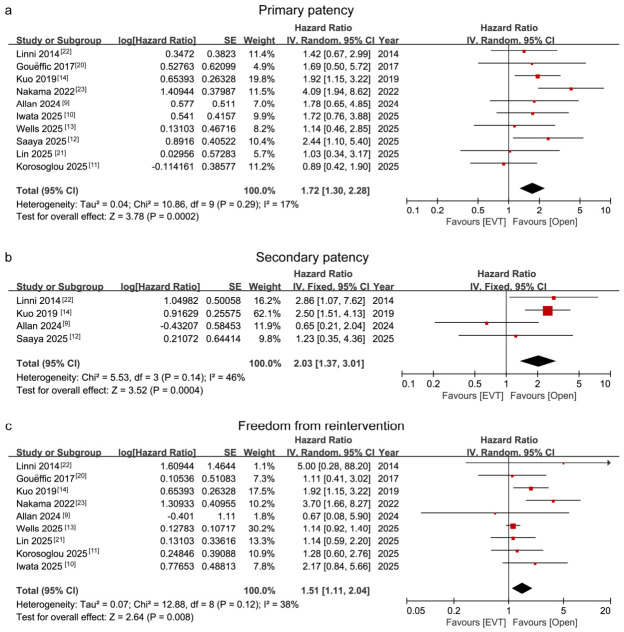
(**a**): Forest plot of time-to-event analysis for loss of primary patency between EVT and open surgery. (**b**): Forest plot of time-to-event analysis for loss of secondary patency between EVT and open surgery. (**c**): Forest plot of the meta-analysis of freedom from reintervention between EVT and open surgery. IV, inverse variance; CI, confidence interval; SE, standard error [9,10,11,12,13,14,20,21,22,23].

Ten studies [9,10,11,12,14,20,21,22,23,24] reported time-to-event data for primary patency and pooled analysis demonstrated that the EVT group had worse primary patency compared with the OS group (EVT vs. OS: HR = 1.72; 95% CI: 1.30–2.28; *p* < 0.001; I^2^ = 17%; Table 5; Figure 7a). Consistent findings were observed in the RCT, cohort study, and hybrid subgroup analyses (Supplementary Tables S1–S3). Four studies [9,12,14,22] reported time-to-event data for secondary patency and synthesized analysis indicated that the EVT group had worse secondary patency compared with the OS group (EVT vs. OS: HR = 2.03; 95% CI: 1.37–3.01; *p* < 0.001; I^2^ = 46%; Table 5; Figure 7b). Consistent findings were observed in the cohort study subgroup analyses (Supplementary Table S1). Nine studies [9,10,11,13,14,20,21,22,23] provided time-to-event data for freedom from reintervention and pooled analysis revealed that the EVT group was associated with a higher hazard of reintervention rates than the OS group (EVT vs. OS: HR = 1.51; 95% CI: 1.11–2.04; *p* = 0.008; I^2^ = 38%; Table 5; Figure 7c). Five studies [9,10,11,13,23] documented time-to-event data for amputation-free survival and pooled analysis showed no significant difference between the two groups (HR = 1.01; 95% CI: 0.83–1.23; *p* = 0.93; I^2^ = 0%; Table 5). Eight studies [9,10,11,12,13,20,22,23] presented time-to-event data for overall survival and no significant difference was observed between the two groups (HR = 1.09; 95% CI: 0.91–1.29; *p* = 0.36; I^2^ = 9%; Table 5).

### 3.5. GRADE Assessment

The certainty of evidence was moderate for 30-day perioperative morbidity, wound complications, surgical-site infection, and loss of primary patency, and low for the remaining outcomes. Moderate-certainty evidence indicated that EVT was associated with lower 30-day perioperative morbidity, fewer wound complications and surgical-site infections, but a higher risk of loss of primary patency. Low-certainty evidence suggested that EVT was associated with fewer lymphatic complications, fewer perioperative myocardial infarctions, and a shorter length of hospital stay, but with higher risks of distal embolization, follow-up major amputation, loss of secondary patency, and reintervention. Detailed GRADE assessments are provided in Supplementary Table S4.

### 3.6. Publication Bias and Sensitivity Analysis

**Figure 8:**
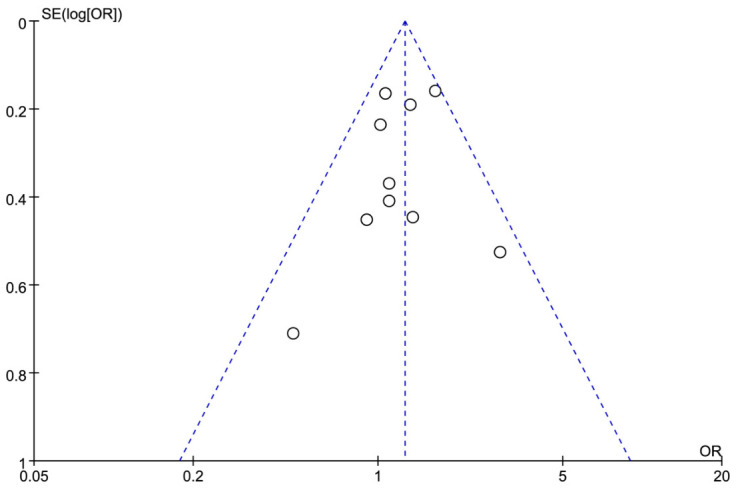
The funnel plot of the meta-analysis of wound complications between EVT and open surgery.

For outcomes including at least 10 studies, potential publication bias was assessed using funnel plots (Figure 8). Visual inspection did not reveal marked asymmetry; however, interpretation was limited by the relatively small number of included studies and the low event rate. For outcomes with substantial heterogeneity (I^2^ > 50%), including length of hospital stay, Post-ABI, CLTI, Rutherford stage > 3, calcification ≥ 50%, reoperation, hematoma, and follow-up myocardial infarction, leave-one-out sensitivity analyses were performed. Sequential exclusion of individual studies did not materially alter the pooled effect estimates (Supplementary Figures S1 and S2).

## 4. Discussion

Common femoral endarterectomy has traditionally been considered the reference standard for CFA occlusive disease because of its durable patency [1,2]. However, evidence regarding the efficacy of EVT in this specific anatomical segment remains relatively limited and controversial. Previous studies [21,22] have primarily focused on the short-term feasibility and minimally invasive appeal of EVT, while the effect of treatment modality on long-term clinical outcomes in CFA atherosclerosis has remained inconsistent. In the present study, although patients in the OS group presented with lower baseline ABI, differences in tobacco use were not statistically significant, and the EVT cohort had a higher baseline burden of coronary artery disease and hyperlipidemia. These findings highlight significant differences in patient selection between the two approaches in real-world practice.

Consistent with prior evidence [6,8], our analysis revealed that EVT was associated with lower short-term perioperative morbidity. The EVT group demonstrated remarkably lower rates of overall complications, wound infections, and lymphatic fistulas, alongside a reduced incidence of myocardial infarction and shorter hospital stays. However, some previous literature [24,25,26,27] has warned of the unique intraoperative risks associated with endovascular interventions in the groin. Aligning with these concerns, our results showed that distal embolization events were significantly more frequent in the EVT group. This complication was likely mechanistically driven by plaque shifting, fragmentation, and embolization during balloon inflation or atherectomy, particularly given that CFA lesions are frequently bulky and heavily calcified. Conversely, the distinct advantage of EVT in reducing wound-related complications and lymphatic fistula is naturally explained by the avoidance of surgical incisions in the groin, an area notoriously prone to poor healing and infection.

Regarding long-term durability, this study found that open surgery was associated with more durable patency during follow-up, whereas some recent small-scale reports have suggested that endovascular approaches might be sufficient for certain lesion types. Our pooled time-to-event analyses demonstrated that the open surgery group had superior primary patency, secondary patency, and freedom from reintervention. This durability may reflect the ability of endarterectomy to remove bulky calcified plaque and preserve the femoral bifurcation, although this mechanism cannot be confirmed from aggregate-level data. The CFA is an anatomically unique segment subjected to repetitive flexion, extension, and torsional forces during routine mobility, which can compromise the structural integrity of endovascular devices and predispose patients to intimal hyperplasia and restenosis [28,29,30]. However, these superior long-term outcomes must be interpreted with caution, as patients in the open surgery group had a lower baseline prevalence of hyperlipidemia and coronary artery disease, which are established systemic risk factors for atherosclerotic disease progression. Overall, current evidence suggests that open surgery provides a more durable hemodynamic result; however, further well-designed prospective studies adjusting for these baseline covariates are necessary to confirm these findings.

Our follow-up results demonstrated that the open surgery group achieved a significantly lower amputation rate than the EVT group, whereas several previous studies [31,32,33] reported no significant difference in limb salvage between the two modalities. EVT might inadvertently increase the risk of recurrent limb-threatening ischemia through higher rates of restenosis and the aforementioned distal embolization events, rather than providing a lasting revascularization solution. The higher observed amputation rate after EVT may reflect differences in lesion complexity, patient selection, restenosis risk, distal embolization, or follow-up duration. It is also important to note that involvement of the profunda femoris artery, femoral bifurcation disease, calcification severity, lesion length, and the status of inflow and outflow vessels may all influence treatment selection, technical success, patency, restenosis, reintervention, and limb-related outcomes. Therefore, although our meta-analysis provides an overall comparison between EVT and OS, the pooled results should be interpreted with caution when applied to individual patient selection. Future studies should standardize the reporting of anatomical characteristics, lesion complexity, device use, and postoperative medical therapy to better define the patient subgroups most likely to benefit from each treatment strategy.

Hybrid procedures also represent an important strategy for the treatment of CFA disease, particularly in patients with CFA lesions combined with multilevel disease involving the iliac, superficial femoral, or infrapopliteal arteries. These procedures typically combine CFA endarterectomy with proximal or distal endovascular intervention, aiming to simultaneously address local femoral disease, bifurcation involvement, and multilevel inflow or outflow lesions. Thus, in real-world practice, hybrid procedures may reflect more complex anatomical disease and greater treatment demands. Simply classifying hybrid procedures as either OS or EVT may introduce methodological heterogeneity and affect the assessment of the efficacy of isolated EVT and isolated OS. To minimize the impact of this factor on our conclusions, we performed a subgroup analysis of hybrid procedures in the supplementary analyses, and the results were generally consistent with the overall findings. However, because the definitions, technical components, lesion extent, and follow-up reporting of hybrid procedures varied across studies, these findings should still be interpreted cautiously. Future studies should adopt more standardized definitions and stratified reporting of hybrid procedures to clarify their true role in the management of CFA disease.

We used the GRADE approach to assess the certainty of evidence for the main outcomes. Because the included studies were predominantly observational and treatment allocation was generally based on clinical judgment rather than randomization, the evidence was downgraded owing to selection bias and baseline confounding. For short-term outcomes, including perioperative complications, wound infection, lymphatic fistula, and length of hospital stay, the direction of effect was relatively consistent despite the inherent limitations of the study designs. These findings are also biologically plausible, given that EVT avoids groin incision and reduces surgical trauma; therefore, their interpretation is relatively robust. In contrast, long-term time-to-event outcomes, such as patency and freedom from reintervention, partly relied on reconstructed Kaplan–Meier data and were influenced by differences in follow-up duration, lesion anatomy, device use, postoperative medical therapy, and endpoint definitions. These factors contributed to indirectness, imprecision, and clinical heterogeneity. For low-event outcomes, such as amputation, the limited number of events further reduced the stability of the effect estimates. Accordingly, our conclusions regarding the long-term durability advantage of OS and the short-term safety benefit of EVT should be interpreted cautiously in light of the certainty of evidence assessed by GRADE.

Overall, the treatment strategy for CFA disease should be individualized according to anatomical characteristics, perioperative risk, and patient-specific factors. EVT may be beneficial for selected patients, particularly frail individuals or high-risk surgical candidates, because it is associated with lower perioperative morbidity and fewer groin wound complications. In suitable surgical candidates, OS may still provide superior long-term durability. Further well-designed prospective studies are warranted, with standardized reporting of anatomical characteristics, lesion complexity, hybrid procedure components, device use, perioperative medical therapy, and long-term follow-up outcomes, to more accurately identify the patient subgroups most likely to benefit from each treatment strategy.

## 5. Limitations

First, detailed lesion characteristics, including lesion severity and involvement of the profunda femoris artery, were not consistently available, which precluded subgroup analyses to further explore potential sources of heterogeneity among the included studies. Second, the specific endovascular devices used were insufficiently reported, limiting our ability to assess the potential advantages of contemporary device-specific EVT strategies. Third, postoperative medical therapy was not described in adequate detail, and therefore the potential impact of differences in antithrombotic or adjunctive treatment regimens on target vessel patency and limb salvage could not be evaluated. Fourth, most pooled estimates were derived from observational studies and unadjusted aggregate data, which limits causal inference. In addition, several HRs were reconstructed from published Kaplan-Meier curves rather than extracted directly, introducing potential measurement error. Finally, social determinants of health, such as race, socioeconomic status, and access to healthcare, were not adequately addressed in the included studies. Despite these limitations, taken together, the available evidence supports OS as the more durable strategy for appropriate surgical candidates, whereas EVT remains a reasonable alternative for selected patients at high surgical risk or with anatomy suitable for endovascular intervention.

## 6. Conclusions

In patients with CFA atherosclerotic occlusive disease, EVT was associated with lower perioperative morbidity, fewer wound-related complications—particularly groin wound complications—and shorter hospitalization, whereas OS was associated with superior long-term durability, including higher primary and secondary patency and lower hazards of re-intervention. Major amputation appeared less frequent after OS in pooled event-rate analyses; however, this finding should be interpreted cautiously given the low event rate, heterogeneity in follow-up duration, and potential confounding. EVT may be beneficial in selected patients, particularly frail patients or those at high surgical risk, in whom reduced perioperative morbidity and fewer groin wound complications are clinically relevant. Nevertheless, treatment selection should remain individualized.

## Figures and Tables

**Table 1:** Characteristics of the included studies.

Study (Year)	Study Interval	Country	Study Type	EVT	OS	Outcomes Reported	Follow-Up (Months) Mean ± SD	Risk-of-Bias Assessment
Linni (2014) [22]	2011–2013	Austria	RCT	40	40	Patency rate; complications; reintervention; limb salvage	10.74 ± 2.64	Some concerns
Gouëffic (2017) [20]	2011–2013	France	RCT	56	61	30-day morbidity; 30-day mortality; reintervention	20.42 ± 4.29	Some concerns
Kuo (2019) [14]	2013–2016	China	Retrospective	40	60	Patency rate; complications; reintervention; limb salvage	23.41 ± 3.29	Moderate risk
Nakama (2022) [23]	2011–2017	Japan	Retrospective PSM	482	212	Patency rate; morbidity; reintervention	11.98 ± 4.18	Moderate risk
Shaker (2023) [24]	2010–2019	Egypt	RCT	18	18	Amputation; infection; patency rates; complications	24.78 ± 2.18	Some concerns
Allan (2024) [9]	2019–2022	Australia	Retrospective	33	55	Patency rate; complications; all-cause mortality	12.25 ± 3.67	Moderate risk
Wells (2025) [13]	2013–2020	USA	Retrospective	218	229	Patency rate; complications	47.16 ± 12.28	Moderate risk
Saaya (2025) [12]	2018–2023	Russia	RCT	31	31	Patency rate; complications; all-cause mortality	32.5 ± 12.1	Some concerns
Lin (2025) [21]	2018–2023	China	Retrospective	11	20	Patency rate; complications	22.05 ± 16.3	Moderate risk
Korosoglou (2025) [11]	2015–2022	Germany	Retrospective	213	613	All-cause mortality; reintervention; amputation-free survival	50.12 ± 8.43	Moderate risk
Iwata (2025) [10]	2018–2020	Japan	Retrospective PSM	101	253	Primary patency; perioperative complications; reintervention; overall survival	12.38 ± 2.43	Moderate risk

EVT: endovascular treatment; OS: open surgery; RCT: randomized controlled trial; PSM: propensity score matching; SD: standard deviation.

**Table 2:** Pooled patient baseline characteristics.

Variables	No. of Studies	No. of Patients	EVT	OS	MD/OR, 95% CI #	*p* Value	I^2^
Age [9,10,11,12,13,14,20,21,22,23,24]	11	2835	1243	1592	0.50 (−0.41–1.42)	0.28	43%
Pre-ABI [11,12,20,21,23,24]	5	1704	780	924	0.02 (0.01–0.04)	0.002	27%
Diabetes [9,10,11,12,13,14,20,21,22,23,24]	11	2835	1243	1592	1.11 (0.85–1.45)	0.44	48%
Hyperlipidemia [9,10,12,13,14,20,21,22,24]	9	1787	660	1127	1.43 (1.14–1.79)	0.002	0%
Gender (male) [9,10,11,12,13,14,20,21,22,23,24]	11	2835	1243	1592	0.86 (0.65–1.14)	0.30	45%
Hypertension [10,12,13,14,20,21,22,24]	8	1699	627	1072	1.07 (0.81–1.40)	0.64	0%
Tobacco use [9,10,11,12,13,14,20,21,22,23,24]	11	2835	1243	1592	0.80 (0.61–1.05)	0.11	40%
Chronic renal insufficiency [9,10,11,13,14,20,21,23]	8	2657	1154	1503	1.21 (0.98–1.49)	0.08	0%
Rest pain [9,10,11,12,13,14,20,21,23]	9	2308	985	1323	1.18 (0.86–1.61)	0.31	26%
CLTI [9,10,11,13,14,20,21,23]	8	1911	981	930	0.88 (0.56–1.39)	0.58	63%
Hemodialysis [10,13,14,20]	4	1018	415	603	0.97 (0.67–1.41)	0.87	0%
Rutherford category > 3 [9,10,11,12,14,20,21,22,23,24]	10	2388	1025	1363	0.89 (0.61–1.31)	0.55	63%
History of stroke [9,10,12,13,14,21,23,24]	8	1812	934	878	0.97 (0.75–1.25)	0.81	0%
Calcification ≥ 50% [10,12,14,20,21,23]	6	1358	721	637	0.89 (0.70–1.13)	0.34	58%
Claudication [9,10,12,21,23,24]	6	1265	676	589	0.93 (0.69–1.25)	0.63	25%
CAD [9,10,11,12,13,14,20,22,23,24]	10	2804	1232	1572	1.26 (1.07–1.48)	0.005	9%

EVT: endovascular treatment; OS: open surgery; CI: confidence interval; ABI: ankle brachial index; CLTI: chronic limb threatening ischemia; CAD: coronary artery disease; OR: odds ratio; MD: mean difference. #: MDs were used for continuous variables and ORs for dichotomous variables.

**Table 3:** Pooled 30-day postoperative outcomes.

Variables	No. of Studies	No. of Patients	EVT	OS	MD/OR, 95% CI #	*p* Value	I^2^
LOS [9,10,11,12,13,14,20,21,23]	9	2719	1185	1534	−4.68 (−5.49–−3.86)	<0.001	89%
Post-ABI [11,20,21,23,24]	5	1704	780	924	−0.04 (−0.11–0.03)	0.29	96%
Perioperative morbidity [9,10,11,12,14,20,21,22,23,24]	10	2799	1225	1574	0.34 (0.26–0.44)	<0.001	0%
Perioperative mortality [11,12,13,14,21,22,23]	7	2532	1105	1427	1.16 (0.54–2.46)	0.70	10%
Reoperation [9,10,11,12,13,22]	7	2551	1118	1433	0.51 (0.17–1.52)	0.23	56%
Wound complications [9,10,11,12,14,20,21,22,23,24]	10	2799	1225	1574	0.14 (0.09–0.23)	<0.001	0%
New hemodialysis [9,10,12,13,14,20,21,22,23]	5	1360	784	576	1.21 (0.66–2.22)	0.53	0%
Distal embolization [10,11,12,13,14,20,21,22,23]	9	2711	1192	1519	2.45 (1.28–4.70)	0.007	48%
Major amputation [9,12,13,21,23]	5	1322	775	547	0.78 (0.31–1.99)	0.61	36%
Hematoma [10,12,13,14,20,21,22]	7	1191	497	694	0.71 (0.39–1.31)	0.28	51%
Surgical site infection [10,11,13,14,20,21,22,23]	8	2706	1183	1523	0.11 (0.05–0.22)	<0.001	0%
Lymphatic fistula [10,11,12,14,20,22,23]	7	2233	963	1270	0.08 (0.03–0.25)	<0.001	0%
Stroke [10,12,13,14,20,21,22,24]	8	1227	515	712	0.97 (0.36–2.59)	0.95	0%
Myocardial infarction [11,13,14]	3	1373	471	902	0.17 (0.04–0.72)	0.02	0%

EVT: endovascular treatment; OS: open surgery; CI: confidence interval; LOS: length of hospital stay; ABI: ankle brachial index; OR: odds ratio; MD: mean difference; #: MDs were used for continuous variables and ORs for dichotomous variables.

**Table 4:** Pooled follow-up outcomes.

Variables	No. of Studies	No. of Patients	EVT	OS	OR, Random, 95% CI	*p* Value	I^2^%
Mortality [9,10,11,12,13,21,22]	7	1888	647	1241	1.48 (0.75–2.95)	0.26	38%
Myocardial infarction [10,12,13,14,22]	5	1807	612	1195	0.56 (0.12–2.63)	0.46	53%
Major amputation [9,10,11,12,13,14,21,22]	8	1988	687	1301	2.42 (1.19–4.93)	0.02	0%
Reintervention [9,12,13,21]	4	628	293	335	1.22 (0.87–1.71)	0.26	0%
Stroke [10,11,12,13,14,21,22]	7	1900	654	1246	1.06 (0.46–2.46)	0.89	34%

EVT: endovascular treatment; OS: open surgery; OR: odds ratio; CI: confidence interval.

**Table 5:** Pooled time-to-event outcomes.

Variables	No. of Studies	No. of Patients	Hazard Ratio, 95% CI	*p* Value	I^2^%
Primary patency [9,10,11,12,14,20,21,22,23,24]	10	2799	1.72 (1.30–2.28)	<0.001	17%
Secondary patency [9,12,14,22]	4	330	2.03 (1.37–3.01)	<0.001	46%
Amputation-free survival [9,10,11,13,23]	5	2409	1.01 (0.83–1.23)	0.93	0%
Freedom from reintervention [9,10,11,13,14,20,21,22,23]	9	2737	1.51 (1.11–2.04)	0.008	38%
Overall survival [9,10,11,12,13,20,22,23]	8	2668	1.09 (0.91–1.29)	0.36	9%

CI, confidence interval.

## Data Availability

The data supporting the findings of this study are available from the corresponding author upon reasonable request.

## Supplementary Materials

The following supporting information can be downloaded at: https://www.mdpi.com/article/10.3390/jcm15145353/s1, Figure S1: Leave-one-out sensitivity analysis in length of hospital stay, CLTI, Rutherford stage > 3 and calcification ≥ 50%; Figure S2: Leave-one-out sensitivity analysis in post-ABI, reoperation, hematoma, and follow-up myocardial infarction; Table S1: Meta-analysis results for the cohort subgroup; Table S2: Meta-analysis results for the RCT subgroup; Table S3: Meta-analysis results for the hybrid subgroup; Table S4: GRADE evidence profile; Table S5: PRISMA checklist.
