# Supplementary material for: Endovascular Therapy Versus Open Surgery for Common Femoral Artery Atherosclerotic Occlusive Disease: A Systematic Review and Meta-Analysis
Source: J Clin Med. 2026 Jul 8;15(14):5353. doi: 10.3390/jcm15145353 (PMC13412851; doi:10.3390/jcm15145353)
Supplement: Supplementary file 1 [file jcm-15-05353-s001.zip › Supplementary Table S1-S4.pdf]

Journal of
Clinical Medicine

MDPI

Supplementary Table S1. Meta-analysis results for the Cohort subgroup

Outcome	Studies	EVT	OS	MD/OR, 95% CI[#]	P value	I²
Length of stay[9-11,13,14,21,23]	7	1098	1442	-5.01 (-5.98– -4.03)	<0.001	91.2%
Preoperative ABI[11,21,23]	3	706	845	0.00 (-0.04–0.05)	0.843	63.3%
Hyperlipidemia[9,11,13,14,21]	5	515	977	1.54 (1.19–1.99)	<0.001	0.0%
Coronary artery disease[9-11,13,14,23]	6	1087	1422	1.28 (1.07–1.51)	0.005	0.0%
Perioperative morbidity[9-11,13,14,21,23]	7	1098	1442	0.34 (0.26–0.45)	<0.001	0.0%
Major amputation	6	434	648	2.64 (1.10–6.33)	0.029	0.0%
Wound complications[9-11,13,14,21,23]	7	1098	1442	0.16 (0.09–0.27)	<0.001	0.0%
Surgical-site infection[9-11,13,14,23]	6	1087	1422	0.12 (0.06–0.26)	<0.001	0.0%
Lymphatic fistula[10,11,14,23]	4	836	1138	0.11 (0.02–0.48)	0.003	0.0%
Freedom from reintervention[9-11,13,14,21,23]	7	-	-	1.31 (1.10–1.55)	0.002	37.9%
Secondary patency[9,14]	2	-	-	2.01 (1.27–3.19)	<0.001	45.7%
Primary patency[9-11,13,14,21,23]	7	-	-	1.72 (1.28–2.30)	<0.001	39.2%

#: MD were used for continuous variables, HR for time-to-event variables and OR for dichotomous variables; MD: mean difference; HR: hazard ratio; CI: confidence interval; EVT :endovascular treatment; OS:open surgery; Fixed-effect model was used when I² ≤ 50%; random-effects model was used when I² > 50%.

Supplementary Table S2. Meta-analysis results for the RCT subgroup

Outcome	Studies	EVT	OS	MD/OR, 95% CI#	P value	I²
Length of stay[12,20]	2	87	92	-3.56 (-4.31– -2.82)	<0.001	28.5%
Preoperative ABI[12,20]	2	74	79	0.02 (-0.12–0.15)	0.811	0.0%
Hyperlipidemia[12,20,22,25]	4	145	150	1.04 (0.63–1.72)	0.881	0.0%
Coronary artery disease[12,20,22,24]	4	145	150	1.15 (0.72–1.84)	0.548	38.3%
Major amputation[12,22]	2	253	653	1.24 (0.19–8.35)	0.822	0.0%
Lymphatic fistula[12,20,22]	3	127	132	0.20 (0.03–1.15)	0.071	0.0%
Freedom from reintervention[20,22]	2	-	-	1.31 (0.51–3.37)	0.578	0.0%
Secondary patency[12,22]	2	-	-	2.08 (0.96–4.52)	0.06	5.0%
Primary patency[12,20,22]	3	-	-	1.81 (1.10–2.97)	0.020	0.0%

＃: MD were used for continuous variables, HR for time-to-event variables and OR for dichotomous variables; MD: mean difference; HR: hazard ratio; CI: confidence interval; EVT :endovascular treatment; OS:open surgery; Fixed-effect model was used when $I^2 \leq 50\%$; random-effects model was used when $I^2 > 50\%$.

Supplementary Table S3. Meta-analysis results for the Hybrid subgroup

Outcome	Studies	EVT	OS	MD/OR, 95% CI#	P value	I²
Length of stay[11,12,21]	3	255	664	-4.34 (-4.53– -4.14)	<0.001	0.0%
Preoperative ABI[11,21,24]	3	242	651	-0.01 (-0.05–0.03)	0.600	13.0%
Hyperlipidemia[11,12,21,24]	4	273	682	1.81 (1.30–2.53)	<0.001	0.0%
Coronary artery disease[11,12,24]	3	262	662	1.53 (0.75–3.10)	0.240	51.7%
Perioperative morbidity[11,12,21]	3	255	664	0.29 (0.18–0.47)	<0.001	0.0%
Major amputation[12,21]	2	42	51	1.07 (0.18–6.16)	0.94	0.0%
Wound complications[11,12,21]	3	255	664	0.11 (0.04–0.26)	<0.001	0.0%
Lymphatic fistula[11,12]	2	244	644	0.06 (0.01–0.45)	0.007	2.9%
Freedom from reintervention[11,21]	2	-	-	1.20 (0.73–1.97)	0.478	0.0%
Primary patency[11,12,21]	3	-	-	1.35 (0.82–2.21)	0.233	43.0%

#: MD were used for continuous variables, HR for time-to-event variables and OR for dichotomous variables; MD: mean difference; HR: hazard ratio; CI: confidence interval; EVT :endovascular treatment; OS:open surgery; Fixed-effect model was used when $I^2 \leq 50\%$; random-effects model was used when $I^2 > 50\%$.

Supplementary Table S4: GRADE evidence profile

outcome	No. of studies	MD/OR, 95% CI[#]	Risk of bias	Inconsistency	Indirectness	Imprecision	Publication bias	Overall certainty
30-day perioperative morbidity	10	0.34 (0.26–0.44)	Serious	Not serious	Not serious	Not serious	Undetected	**Moderate**
Wound complications	10	0.14 (0.09–0.23)	Serious	Not serious	Not serious	Not serious	Undetected	**Moderate**
Surgical-site infection	8	0.11 (0.05–0.22)	Serious	Not serious	Not serious	Not serious	Undetected	**Moderate**
Lymphatic complications	7	0.08 (0.03–0.25)	Serious	Not serious	Not serious	Serious	Undetected	**Low**
Perioperative myocardial infarction	3	0.17 (0.04–0.72)	Serious	Not serious	Not serious	Serious	Unable to assess	**Low**
Length of hospital stay	9	-4.68(-5.49 - -3.86)	Serious	Serious	Not serious	Not serious	Undetected	**Low**
Distal embolization	9	2.45 (1.28–4.70)	Serious	Not serious	Not serious	Serious	Undetected	**Low**
Follow-up major amputation	8	2.42 (1.19–4.93)	Serious	Not serious	Not serious	Serious	Unable to assess	**Low**
Loss of primary patency	10	1.72 (1.30–2.28)	Serious	Not serious	Not serious	Not serious	Undetected	**Moderate**
Loss of secondary patency	4	2.03 (1.37–3.01)	Serious	Not serious	Not serious	Serious	Unable to assess	**Low**
Reintervention	9	1.51 (1.11–2.04)	Serious	Not serious	Not serious	Serious	Undetected	**Low**

＃: MD were used for continuous variables, HR for time-to-event variables and OR for dichotomous variables; MD: mean difference; HR: hazard ratio; CI: confidence interval
