# Supplementary figures and images for: Endovascular Therapy Versus Open Surgery for Common Femoral Artery Atherosclerotic Occlusive Disease: A Systematic Review and Meta-Analysis
Source: J Clin Med. 2026 Jul 8;15(14):5353. doi: 10.3390/jcm15145353 (PMC13412851; doi:10.3390/jcm15145353)

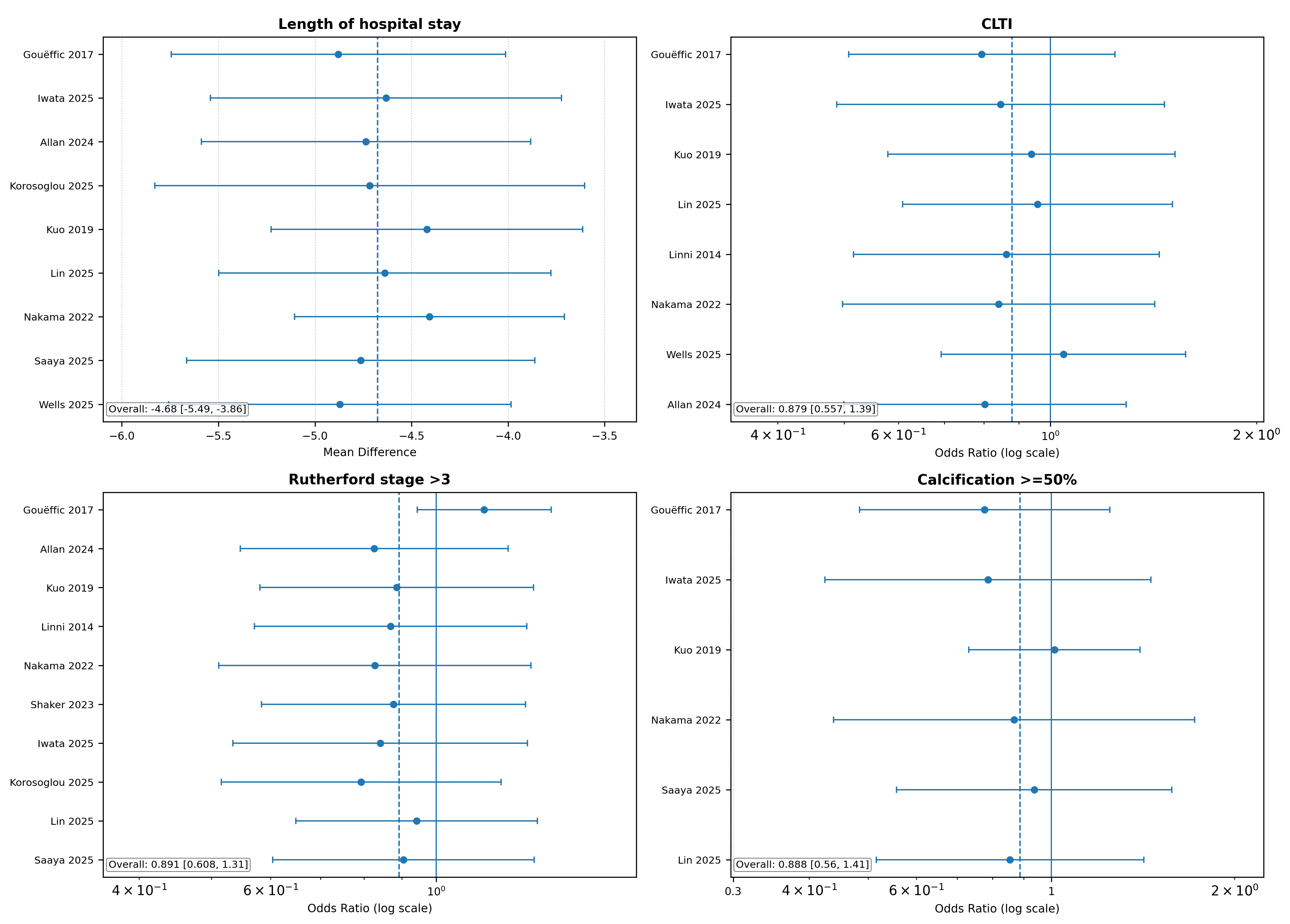

Supplement: Supplementary file 1 [file jcm-15-05353-s001.zip › Supplementary Figure S1.png]

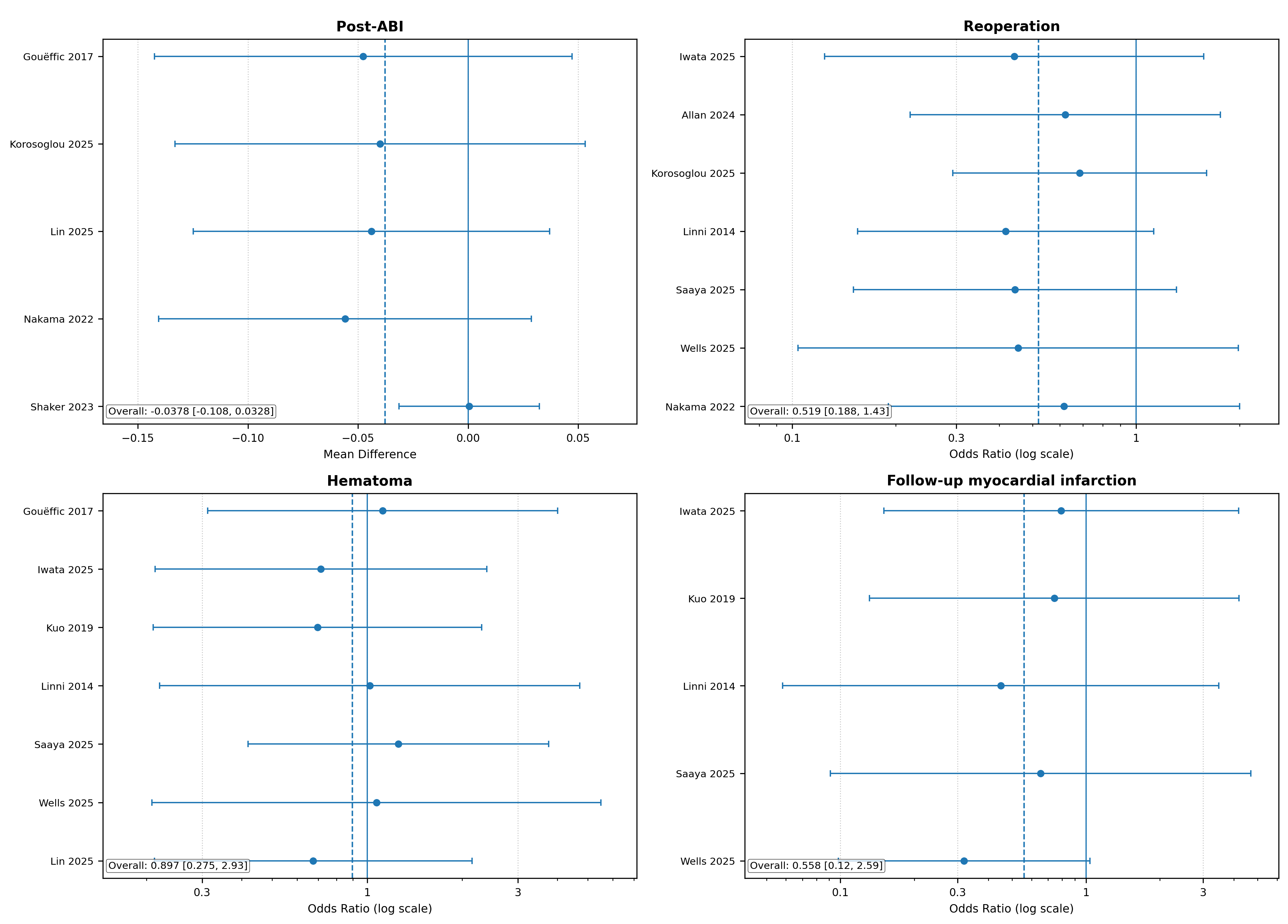

Supplement: Supplementary file 1 [file jcm-15-05353-s001.zip › Supplementary Figure S2.png]
